# Anaesthesia of decapod crustaceans

**DOI:** 10.1016/j.vas.2022.100252

**Published:** 2022-05-14

**Authors:** Cecília de Souza Valente

**Affiliations:** Bio-Resources Unit, National University of Ireland Galway, University Road, Galway H91TK33, Ireland

**Keywords:** Anaesthetics, Analgesia, Invertebrates, Nociception, Pain, Sedation

## Abstract

Decapod crustaceans (crabs, hermit crabs, lobsters, crayfish, shrimps, prawns) are sentient beings, not only responding to noxious stimuli but also being capable of feeling pain, discomfort, and distress. General anaesthesia aims at producing analgesia, immobilization, and unconsciousness, while sedation reduces consciousness, stress, and anxiety, though without analgesia. Anaesthesia is recommended to ensure animal welfare and suppress nociception, pain, and suffering in painful and distressing practice that impairs decapods’ welfare. These include long term restrain, surgical procedures, pain control, examination, diagnostic, sampling, treatment, transportation, and euthanasia. The necessary anaesthetic depth, from sedation to surgical anaesthesia, depends on the procedure type. Anaesthetic bath and injection are commonly used, besides inhalation, local anaesthesia, and intracardiac injection. Agents used for the anaesthetic bath include eugenol, isoeugenol, lidocaine, halothane, and essential oils of lemon balm, lemongrass, lemon verbena, and sandalwood. While alphaxalone, eugenol, ketamine-xylazine, lidocaine, morphine, procaine, tiletamine-zolazepam, and xylazine can be used as injectable agents administered on the arthrodial membrane or intramuscular injection. Halothane can be used on inhalation anaesthesia. Local anaesthetics include lidocaine and benzocaine. Notwithstanding, many others are detrimental or ineffective to decapods, thus discouraged. They include but not limited to hypothermia, carbon dioxide, chlorpromazine, chloroform, ethanol, ether, magnesium salts, tricaine methanesulfonate (MS-222), mint and lavender essential oils, passionflower extract, and valerian. Decapods’ welfare, protection, and veterinary attention should not be neglected, but they must receive ethical treatment, including the best of our knowledge and available tools to ensure they are free of pain and discomfort whenever we deal with them.

## Highlights

• Decapods have nociceptive response and can also experience pain and distress.
• General anaesthesia produces analgesia, immobility, and unconsciousness.
• Decapods commonly are anaesthetised via injection or anaesthetic bath.
• Sedation is used for mild procedures, e.g., transportation.
• Decapods’ right to be free of pain must be safeguarded.

## Graphical abstract

**Figure:**
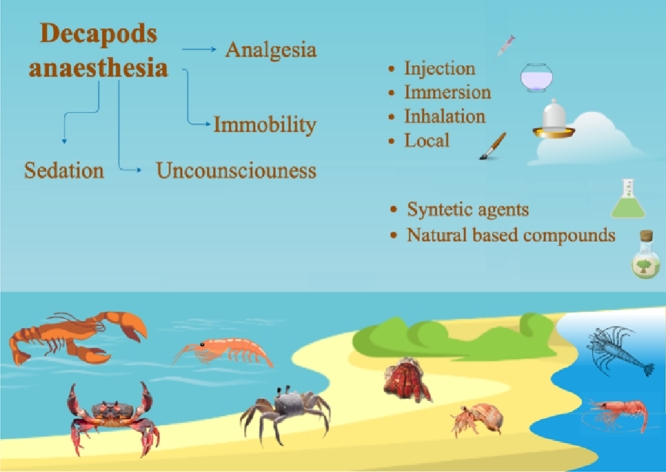

## 1 Introduction

The last years were acknowledged by the debate and progress on Animal Sentience and Welfare. As an example, in 2021, the UK Government officially recognized the sentience of all decapod crustaceans and these animals are now included on the Animal Welfare (Sentience) Bill (Animal Welfare (Sentience) Bill [HL], 2022). It is thus proof of veterinary excellence, high-quality science, and respect for animal ethics to ensure superior welfare standards in all practices and procedures involving animals. This includes the use of anaesthetic and analgesic methods for invertebrates such as decapod crustaceans for the contingent of any possible or potential suffering, discomfort, distress, or pain. It is a core part of the veterinary and animal technician practice to monitor and ensure all aspects of animal health and welfare are granted and protected, including any adverse stimuli on invertebrate animals, even if still not legally required worldwide.

From the Greek *an*, i.e., without, and *aisthesis*, i.e., perception (Schulman & Strichartz, 2012), anaesthesia is, per definition, “a state of controllable, reversible insensibility in which sensory perception and motor responses are both markedly depressed” (Flecknell, 2008, p.382). In other words, a depression of the nervous system where the perception of sensations and responses to them are suppressed (Wounden & Miller, 2012). While analgesia is the temporary inhibition or diminution of pain perception through action on specific nociceptors and nervous system, interrupting pain transmission (Flecknell, 2008; Schulman & Strichartz, 2012), and is one of the desirable effects of anaesthesia (Wounden & Miller, 2012). Nociception is the neural process of detection and encoding of noxious stimuli (i.e., actual or potential tissue-damaging stimuli) by nociceptors, the primary sensory neurons transducing and encoding the noxious stimuli (Birch et al., 2021; Griffin & Woolf, 2012; IASP, 2021; Schulman & Strichartz, 2012). Pain is, per definition, “an unpleasant sensory and emotional experience associated with, or resembling that associated with, actual or potential tissue damage” and, differently from nociception, it is not possible to infer pain only by the nociceptors' activity (IASP, 2021). Sedation is the state of reduced consciousness achieved with the use of sedative medication that relieves stress and anxiety, reduces fear, and cause drowsiness, though the response to pain is still present (Flecknell, 2008; Morita et al., 2002; Wolfensohn & Lloyd, 2013). Tranquilizer medication produces relaxing effects without sedation (Flecknell, 2008). Neuromuscular blocking agents or muscle relaxants cause immobility without depression of the nervous system, that is, the paralysation occurs without any analgesic or narcotic effect. Neuromuscular blocking agents must always be administered with proper anaesthesia and analgesia, ensuring total unconsciousness of the animal whenever they are used (Kastrup et al., 2005; Wolfensohn & Lloyd, 2013). Finally, induction of anaesthesia is the process, measured in time, through the different anaesthetic stages, from the awake state until reaching the desired anaesthesia depth, i.e., the anaesthetised state. The recovery of anaesthesia is the restoration of the awake state when recovering from the anaesthetised state after ceasing the anaesthetic agent administration.

Knowing the physiology, anatomy, normal behaviour, and particular necessity of each species is paramount to diligent and proficient anaesthetic practice. Around 80% of the decapod crustaceans are marine animals, while 20% inhabit freshwater, hypersaline, or terrestrial habitats (Anger, 2016). They include crabs and marine and terrestrial hermit crabs, lobsters and crayfish, and shrimps and prawns. These animals are held as exotic pets, kept in zoos, used in scientific research, and raised for human and animal consumption. Decapods are arthropods and, as so, have jointed limbs, chitinous skeleton, are ectothermic, and have open circulation being haemolymph the fluid that circulates in the vessels and bathes the internal organs (Cooper, 1998; Martin & Hose, 2014). Their nervous system comprises the brain, also known as supraesophageal ganglia, which is the main dorsal ganglia and forms the central nervous system. From the brain emerge two pairs of giant axons that connect to the ventral nerve cord, which is composed of nerve fibres of different diameters. The central nervous system is formed by three primary lobes, the deutocerebrum (chemosensory information), the protocerebrum (visual information), and the tritocerebrum (mechanosensory information) (Martin & Hose, 2014; Sandeman et al., 1993). Two eyestalk ganglia are part of the protocerebrum, which forms the optic lobes (Martin & Hose, 2014). Acetylcholine and noradrenaline are neurotransmitters of decapods. Additionally, 5-hydroxytryptamine (i.e., serotonin), glutamate, γ-aminobutyric acid (GABA), dopamine, histamine, norepinephrine, and peptide like-substances are also neurotransmitters observed in decapods (Atwood, 1982; O'Brien, 2008). Harlıoğlu et al. (2020) show a comprehensive list of their neurotransmitters. Decapods may respond differently to anaesthetics in comparison to other animals, as their synaptic receptors may be differently affected or even not affected by the anaesthetics as in other invertebrates (O'Brien, 2008).

Decapods do not only respond to noxious stimuli, but they also have complex cognitive and learning abilities, sentience, memory, and can experience suffering, anxiety, fear, stress, and pain (Elwood, et al., 2009), including crabs (Davies et al., 2019, Elwood & Adams, 2015) and hermit crabs (Appel & Elwood, 2009), lobsters (Casares et al., 2005) and crayfish (Fossat et al., 2014), shrimps (Takahashi, 2021) and prawns (Barr et al., 2008). Signs of pain in decapods include protective responses, rubbing and overgrooming of the affected area, shaking of sore appendage, avoidance and escape response, autotomy, tensioned and rigid body, and muscle spasms (Gardner, 1997; Sneddon, et al., 2014). Since all sentient beings should receive direct moral consideration, that is, the good of the sentient beings matters in itself (Cunha, 2021), potential or actual negative experiences, such as pain, distress, suffering, or anxiety, should be controlled and avoided. In this context, analgesia and anaesthesia are recommended for decapod crustaceans to minimize fear and stress, as well as to control and suppress nociception, pain and suffering during any painful and/or distressing practice or procedure. This includes immobilization associated with analgesia for better animal examination, diagnostic, and sampling (e.g., gill biopsies, haemolymph collection, oral swabs, imaging); long term restrain; practices that impact animal normal behaviour; surgical procedures (e.g., probes implantation, invasive procedures, repair of damaged exoskeleton or wound, amputation); reduction of pain and stress on postoperative or post-procedure; animal handling and transportation; animal treatment; and euthanasia (Cooper, 2011; Pellet & O'Brien, 2019; Quesada et al., 2011; Sladky et al., 2014).

Thereby, this review summarizes the current knowledge on decapod anaesthesia, presenting the different known anaesthetic agents, routes, and doses, and describes the anaesthetic stages on decapods. It discusses recommended pre and post anaesthetic care and addresses discouraged agents. Lastly, the review suggests research areas with knowledge gaps and closes with final considerations.

## 2 Methodology

The methodology was based on the Preferred Reporting Items for Systematic Reviews and Meta-Analyses (PRISMA) guidelines (Moher et al., 2010). The lack of technical information and absence of literary sources that summarize and present the best, currently available options of anaesthetic agents and procedures for decapod crustaceans were at the origin of the research question here reviewed and discussed. With the aim of presenting, summarizing, and debating the research outcomes and currently used and proposed anaesthetics for decapods and related procedures, the literature review started on November 2021 and continued until March 2022. Sources of information and databases used include NCBI – National Center for Biotechnology Information, Science Direct, SciELO – Scientific Electronic Library Online, Google Scholar, NUI Galway Library online database, BDTD - Brazilian Digital Library of Theses and Dissertations, personal library, and personal communication with authors and companies, via *e*-mail, to have access to additional and complementary information, when needed. To perform the literature search, terms searched encompassed anaesthesia of decapod crustaceans; anaesthesia of invertebrates; sedation of decapod crustaceans; sedation of invertebrates; analgesia of decapod crustaceans; analgesia of invertebrates; operative care of decapod crustaceans; operative care of invertebrates; and their equivalent in American English, Portuguese, and Spanish. Decapod crustacean was substituted for crab, hermit crab, lobster, crayfish, shrimp, and prawn, in additional search. Covered dates started with recent literature, from 2017 onwards, and then from 2012 onwards; due to sparsely available studies and to have access to a broader range of studies, no year limit was used to conclude the literature review. Literature search covered review articles, research articles, academic books and book chapters, and academic thesis and dissertations, preferably in English, but also in Portuguese and Spanish - as long one of the abstracts is in English, except for academic books. Criteria for inclusion were clarity and accuracy in methodology; clarity and accuracy in the results throughout the text; positive and negative results, i.e., literature that suggest anaesthetic agents and/or discourage the use of anaesthetic agents, based on its results. Exclusion criteria were vivisection or the use of isolated parts of the animals’ body, e.g., exposed nerve, extracted nerves, isolated axons; confusing or inconsistent data throughout the text; articles not written in English, Portuguese, or Spanish, even if the abstract was written in these languages; conference abstracts. Finally, for easy identification by the reader, section 4.1 highlights (in bold) the four anaesthetic stages, while sections 4.3, 4.4, and 4.5 highlight (in bold) anaesthetic agents and compounds; and section 4.5 highlights (in bold) discouraged anaesthetic agents and compounds.

## 3 Pre-anaesthetic care and attention

### 3.1 Health examination and animal care

All animals should be fully examined before anaesthesia to assess their health condition. Anamnesis and clinical examination include knowing the animal history, prior environmental conditions, nutritional status, and current medication or treatment, searching for external damage, erosion, darkening, or lesions, using magnification, if needed. Including carapace, abdomen, limbs, and head, physiological signs (inc. respiratory condition, heart rate, and muscle tone), observation of behaviour and psychological status (inc. level of activity, feeding behaviour, swimming pattern, body posture, anorexia, prostration, lethargy, hyperexcitement), and any additional condition beyond the one which is leading the animal to undergo anaesthesia (Cooper, 2011; Noga et al., 2020; O'Brien, 2008). Animals with any deteriorated clinical condition, such as unhealthy, injured, and malnourished, may not tolerate or may respond inappropriately to the anaesthesia and present an inadequate recovery (Cooper, 2011). Sick or injured animals should not be anaesthetised unless the purpose of the anaesthesia is for veterinary treatment. During the health examination, the animal should be weighed for the calculation of the correct anaesthetic dose (Cooper, 2004). Animals close to or under moulting should not be anaesthetised, as the anaesthesia interrupts the moulting process and lead to animal death (O'Brien, 2008). A period of acclimation and recovery is recommended if the animal was recently transported or brought to a different environment (Flecknell, 2008).

Noteworthy, the heart rate of decapod crustaceans varies to some degree between species as well as among individuals of the same species and even on the same individual animal (Table 1). They also present temporary periods of acardia (i.e., temporary cardiac pausing) and apnoea (DeFur, 1988; McGaw & Nancollas, 2021). For this reason, McGaw & Nancollas (2021) advise using the heart rate with caution if examined as a single clinical parameter and, ideally, use this physiological indicator associated with a coefficient of variation, taking into consideration the acardia periods. The authors also present crucial recommendations when assessing the heart rate of decapod crustaceans, such as long-term animal acclimation, restriction of animal movement but without immobilization, and continuous heartbeat monitoring. To monitor the heart rate of decapods, practitioner benefits from electrocardiogram (ECG) pads or Doppler probes placed on the carapace above the heart (Noga et al., 2020; Quesada et al., 2011). Noteworthy, decapods have a neurogenic heart, i.e., decapod heart rate is driven by the cardiac ganglion via nerve impulse, though also influenced by the central nervous system and neurohormones (McGaw & Nancollas, 2021). The decapod heart thus can be used as a bio index to assess the effect of anaesthetic agents (Wycoff et al., 2018) and the heartbeats indicate neuronal function.

**Table 1:** Average heartbeat of adult, healthy decapod crustaceans.

**Species**	Beats per minute	Acardia time	Condition	Refs.
**Crab**				
*Cancer irroratus* Say, 1817	91.0 ± 0.9	59.6 ± 7.2 s	Unrestrained	McGaw & Nancollas, 2021
*Cancer maenas* Linnaeus, 1758	79.3 ± 1.2	27.6 ± 0.9 s		
*Cancer magister* Dana, 1852	91.8 ± 1.3	28.5 ± 2.1 s		
*Cancer gracilis* Dana, 1852	65.6 ± 1.4	46.4 ± 2.8 s		
*Cancer productus* Randall, 1840	99.0 ± 1.2	83.8 ± 5.1 s		
*Poppiana dentata* Randall, 1840	83.0 ± 28.0 26° – 32 °C^†^ 74.0 ± 14.0 26° – 30 °C^†^ 70.0 ± 16.0 26 °C^†^	N/C	Unrestrained	Singh et al., 2019
**Lobster**				
*Homarus ammericanus* Edwards, 1837	54.0 ± 13.0 Resting 90.0 ± 5.0 Exercising	N/C	Unrestrained	Guirguis & Wilkens, 1995
*Panulirus japonicus* von Siebold, 1824	82.2 ± 12.6	5 – 15 min 13.8 ± 4.84 BPM Resting	Unrestrained	Yazawa & Katsuyama, 2001
	103.2 ± 19.8	N/C	Restrained	
**Crayfish**				
*Cherax destructor* Clark, 1936	85.27 ± 7.41 20 °C^†^ 141.61 ± 7.98 30 °C^†^	N/C	Unrestrained	Goudkamp et al., 2004
*Procambarus clarkia* Girard, 1852	90.0 ± 31.2^‡^	N/C	Unrestrained	Bini & Chelazzi, 2006
**Shrimp**				
*Neocaridina denticulate* De Haan, 1844	194.0 ± 21.13 Well-nourished	Not commented	Unrestrained	Siregar et al. 2021
	135.2 ± 5.50 Malnourished			
	86.56 ± 15.62Insecticide exposure			
*Penaeus vannamei* Boone, 1931	141 45 ppt^§^ 99 30 ppt^§^ 74 10 ppt^§^	N/C	Restrained	Chen et al. 2016
*Trypaea australiensis* Dana, 1852	100.8 ± 26.21	Decline if anoxia 52.8 ± 19.13 BPM	Unrestrained	Paterson & Throne, 1995
**Prawn**				
*Palaemon adspersus* Rathke, 1836	84 – 240^‡^	Observed	Resting – recently handled	Hagerman & Uglow, 1979

BPM: heartbeats per minute. Sec: seconds. N/C: not commented. ^†^Water temperature. ^‡^Originally measured as beats per second, original values multiplied by 60. ^§^Water salinity.

The requirement of fasting before anaesthesia in decapod is not yet conclusive. Most anaesthetic studies in decapods do not fast their animals. Fasting is recommended for fishes, for a short period (12 to 24 h), to avoid regurgitation and consequent obstruction of the gills with the back warded content (Posner et al., 2019). Notably, decapod crustaceans can regurgitate and vomit (McGaw, 2006, 2007). Obradović (1986); observed crayfish vomiting after inhaling 0.12 vol.% halothane. Matulovic & Oshiro (2016)fasted shrimps for 48 h aiming at keeping ammonia levels low in the water. Despite the lack of specific studies addressing the matter, it is feasible to suggest decapod should be fasted before anaesthesia to avoid regurgitation or vomit, high volume of gastrointestinal content and high nitrogenous compounds on water, thus consequent impairment on animal welfare and gills obstruction. Attention, though, at long fasting period (above 3 days), which impairs decapods’ energy metabolism, leading to depletion of glycogen, lipid and protein reserve on midgut, decreased level of lipid reserve on muscle, increased consumption of oxygen, and high nitrogen excretion (Comoglio et al., 2005; Sacristán et al., 2017).

### 3.2 Environment

Animals should be constantly kept in their optimal environmental conditions during the acclimation, anaesthesia, and recovery time. For terrestrial species, this includes a clean cage as well as ideal temperature and relative air humidity; for aquatic species, clean tank as well as ideal water temperature and salinity. If acclimation and/or anaesthesia is performed in a different water tank than the original one, optimal water parameters (e.g., temperature and salinity) for that species should be achieved and maintained (Ross & Ross, 2009). Water can be from the original animal tank or made fresh. If made fresh, not only water parameters (e.g., temperature, salinity, pH, and hardness) must be the same as the original tank (Cooper, 2011), but one should ensure, far as practicable, that the biological maturity of the water is as close as possible to the original one. Likewise, when working with trapped aquatic crustaceans that can be caught on dry land (e.g., the Chinese mitten crab *Eriocheir sinensis* Edwards, 1853), it is recommended the use of biologically mature water. The water should then be adjusted, during crustacean acclimation, a process that may take several hours to weeks. Notably, abrupt changes in water parameters and conditions (e.g., osmotic stress) trigger crustacean moulting. If the same individual is repeatedly exposed to such a situation, this will lead to animal death due to the inability to go through another moulting.

Anaesthesia should never be performed in a mixture tank, whether of the same species or a different animal species. Each animal should be monitored closely. Attention at water/environmental temperature to do not overheat or cold the animal, always having in mind decapod crustaceans are ectothermic, meaning that their core body temperature is the same as the environment, which influences their metabolic rate (Cooper, 1998). Induction time tends to be shorter in warm weather due to the nature of ectothermic animals (Cooper, 2011). Low temperature leads to low metabolism and decreased respiratory and cardiac rates, while high temperatures may result in hyperventilation. Water oxygenation and filtration help to maintain water quality ((Ross & Ross, 2009)), while water aeration is recommended for all immersion anaesthesia. Attention should be taken if using an aqueous anaesthetic, as the filter may retain it and alter the agent concentration. Likewise, the physicochemical properties of water (e.g., hardness) impact the pharmacokinetics of the anaesthetic agent, thus affecting the ionic regulation and the permeability of cell membranes, consequently influencing the induction time (Cagol, 2020). Additionally, animal anaesthetized using aqueous anaesthetic agents should be removed from the immersion bath soon after the induction, to avoid overexposure. Particularly when using oil agents (e.g., essential oils), which can cover the gills, leading to prolonged exposure to the agent and ventilatory failure. To better help remove the anaesthetic agent from the gills and body surface, animals can be rapidly and thoroughly rinsed with clean water before being placed on the recovery tank (Foley et al., 1966; Matulovic & Oshiro, 2016). Alternatively, the animal can be placed in clean water, hold on to the upright body position, and gently move through the water to help remove the agent. On the contrary, if necessary to prolong the anaesthetic state outside the induction tank, the practitioner can spray water with the anaesthetic over the animal; if necessary to light the anaesthetic state, the practitioner can spray oxygenated water (Cooper, 2001).

Equally, if an aquatic animal is maintained outside water (e.g., for clinical examination, probe installation, or injection), the animal must be placed on a moistened surface, the exoskeleton should be continuously kept damp and never let to dry, and gills supplied with properly oxygenated water (Cooper, 2011; O'Brien, 2008). This can be achieved by constantly spraying freshwater/seawater in the carapace ((Ross & Ross, 2009)); water should contain the anaesthetic agent to maintain the anaesthesia or be oxygenated (and without the anaesthetic) to hasten the recovery time (Cooper, 2011), and be as identical as possible to the original tank, both in terms of water parameters and biological maturity. Oxygenated water is made by pumping pure oxygen into chlorine-free water for 5 min (Cooper, 2011). Noteworthy, despite several aquatic decapods tolerating air exposure, prolonged exposure to air leads to regurgitation, oxidative damage, and death. For example, the Pacific white shrimp *Penaeus vannamei* Boone, 1931 tolerates 10 min of air exposure; after this time, reversible oxidative damage occurs (reversed by water resubmerging) and, after 30 min, death may occur (Liu et al., 2015). Despite Xu et al. (2018) demonstrated *P. vannamei* Boone, 1931 might survive to prolonged air exposure (12 h), extensive oxidative damage occurs, and animal welfare is questionable. Similarly, the American lobster *Homarus americanus* Edwards, 1837 can survive up to continuous three days of air exposure if kept on cold temperature and wet; nevertheless, this stress factor negatively affects lobster physiological status, impairing haemolymph glucose and lactate levels, as well reducing the crustacean hyperglycaemic hormone on lobster eyestalk (Lorenzon et al., 2007). The red rock crab (*Cancer productus* Randall 1840) often regurgitates during aerial exposure (McGaw, 2007). McGaw & Nancollas (2021), however, exposed green crabs (*Cancer* sp. Linnaeus, 1758) to air for 15 min may not present detectable impairments.

Intertidal decapod crustaceans emerge naturally from water. Some species live in the water or closest as possible (e.g., crab *Cancer productus* Randall 1840; hairy shore crab *Hemigrapsus oregonensis* Dana, 1851*;* mud shrimp *Upogebia* sp. Borradaile, 1903); other present normal activity when exposed to air (e.g., crab *Pachygrapsus crassipes* Randall 1840), with gill regulation when burring on sand (e.g., Dungeness crab *Cancer magister* Dana, 1852); or are terrestrial individuals living intertidally (e.g., ghost crab *Ocypode* sp. Fabricius, 1798), while using aerial respiration and with different osmoregulatory states (DeFur, 1988; Morgan et al., 2001; Watson-Zink, 2021). It is of practitioner's responsibility to know the animal biology and physiological need to best direct animal welfare and care during any procedure, including anaesthesia.

### 3.3 Required equipment and safety

Before starting the anaesthetic procedure, the practitioner must ensure all necessary equipment are cleaned and prepared, which include the induction and recovery containers (plastic or glass chamber/tanks with the anaesthetic agent or oxygenated air/water, respectively), necessary procedure tools and instrumentations, balance with a container to weigh the animal, anaesthetic agents, and any additional necessary equipment. All instrumentation and equipment must be properly cleaned and disinfected, being washed with adequate disinfectant (e.g., diluted chlorhexidine), accurately rinsed, and dried between each use (Posner et al., 2019). The practitioner must wear clean gloves and clean clothing/personal protective equipment (e.g., lab coat, scrub suits, surgical gown).

During any handling or restrain, attention should be taken by the practitioner, as some species, particularly hermit crabs and large crabs and lobsters, can cause significant injuries with their pinchers or claws to the handler (Marnell, 2016; Mosley & Lewbart, 2014). Holding them by the cephalothorax helps to prevent being pinched (Zachariah & Mitchell, 2009). Notable, some decapods can be trained to cooperate during handling and observations. Crayfish from the genus *Procambarus* Ortmann, 1905, for example, habituate to being removed from their aquarium and cooperate with the work to be performed, provided they are kept under optimal conditions. Lastly, anaesthetics can also cause allergy or adversely affect the practitioner (e.g., swollen or numbing of hands and arms). Safe handling for both animal and practitioner is imperative.

### 3.4 Anaesthetic plan and records

Designing a proper anaesthetic plan allows the anaesthetist to properly comprehend the anaesthetic protocol, know the expected events, predict unexpected ones, and record the anaesthetic procedures. The anaesthetic plan and records should include, but are not limited to:

• Dated individual records for each animal, including animal's name or ID, and species, and responsible or tutor's name, when applicable.
• Practitioner‘s name and signature.
• Pre-anaesthesia clinical examination and measurements (e.g., weight, sex, age).
• Any medication under course or premedication, including active principle, dose, route of administration, and length of treatment.
• Eventual particularities and needs of the animal species and individual.
• Anticipation of unexpected events or complications and a contingency plan.
• Description of procedure, expected duration, and required anaesthesia depth.
• Chosen anaesthetic agent, dose, active principle, and route.
• Environment/water quality parameters measured during the anaesthetic procedure.
• Time of start, time of end, and duration of anaesthesia.
• Physiological records and anaesthesia monitoring, including timed records of heart rate (when possible), presence or absence of movements and specific reflex. A table to be filled with time and the monitored parameters is particularly useful.
• Description of anaesthesia recovery.
• Post anaesthesia clinical assessment and recommended care.

A.1 Appendix suggests an anaesthetic plan and record for decapod crustaceans that can be used as a guide and tailored for practitioner necessity and reality. An equipment checklist may also be useful to ensure all necessary materials are working and ready for anaesthesia.

Equally, records of all anaesthetic purchases and use should be kept, as well as anaesthetic agents safely stored. This is particularly important for controlled drugs. In this case, accurate accounting records should include commercial drug's name and active principle, date of use, quantity used, quantity in the flask before use, quantity in the flask after use (which can be complemented with before and after weights), the purpose of use, practitioner's name and signature. A.2 Appendix suggests an anaesthetic accountability record.

## 4 Anaesthesia

### 4.1 Anaesthetic stages

**Fig. 1:**
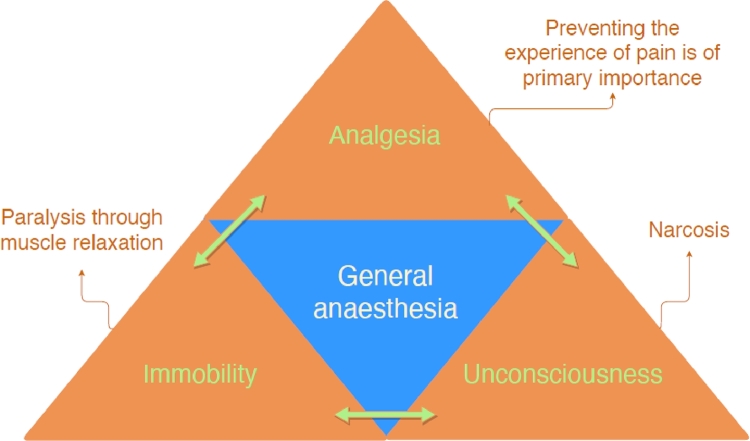
Anaesthetic triad.

**Fig. 2:**
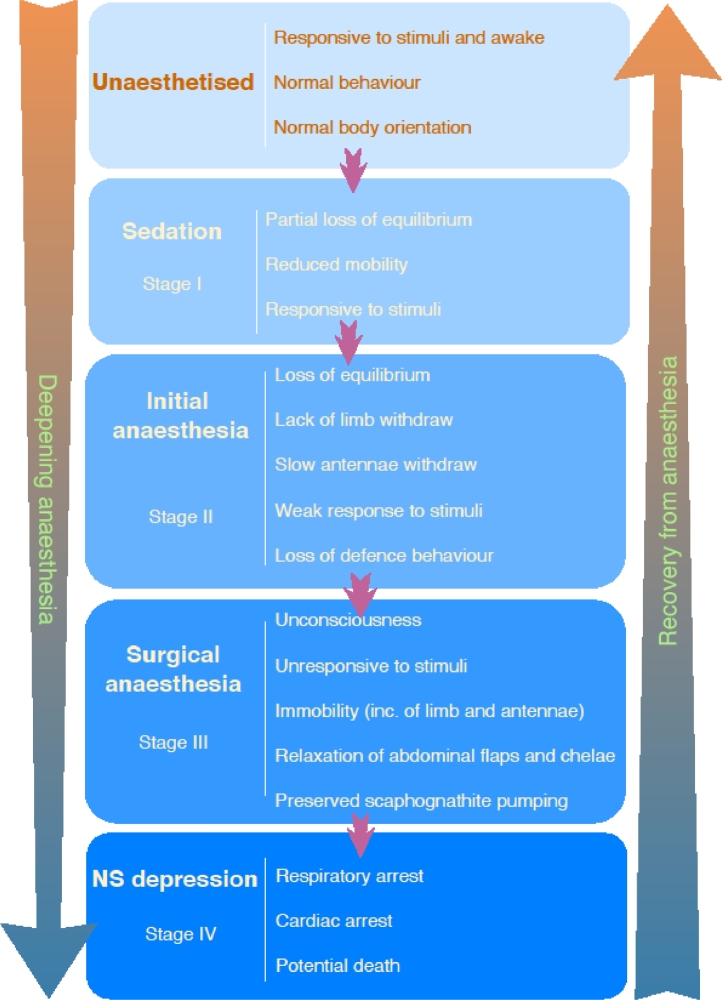
Anaesthetic stages of decapod crustaceans. Based on Coyle et al. (2005), Mosley & Lewbart (2014), Sladky et al. (2014), Wounden & Miller (2012), Wycoff et al. (2018).

Determining the anaesthetic state in decapod crustaceans is difficult, particularly the intermediary stages while deepening anaesthesia. Anaesthetic state includes loss of consciousness, analgesia, amnesia, and immobility, while other desirable effects are muscle relaxation, loss of autonomic reflexes, and anxiolysis (Wouden & Miller, 2012). Considering this, adequate anaesthesia induction in decapods starts with (i) **stage I**: sedation and partial loss of righting reflex and equilibrium, however response to stimuli is still present; excitement stage may also occur (Cowing et al. 2015; Coyle et al., 2005; Wounden & Miller, 2012). (ii) **stage II**: anaesthetic state then evolves to loss of righting reflex and the relaxation of the body, lack of limbs withdraws when stimulated, slowly withdraw of antennae, and loss of defence behaviour (Coyle et al., 2005; Mosley & Lewbart, 2014; Sladky et al., 2014). (iii) **stage III**: total inactivity, including immobility of limbs and antennae, associated with relaxation of abdominal flaps and chelae, lack of eyestalk withdrawal, with maintained scaphognathite pumping, i.e., preserved ventilatory function with gill current, indicate deep anaesthesia (Mosley & Lewbart, 2014; Wycoff et al., 2018). And (iv) **stage IV**: extreme depression of the central nervous system with cardiac and respiratory arrest, with potential death. On vertebrates, stage III comprehends four phases, **phase 1**: light surgical stage; **phase 2**: light to medium surgical stage, the one recommended to be achieved; **phase 3**: medium to deep surgical stage, acceptable but may be too depth and unnecessary; **phase 4**: unrecommended deep surgical stage, considered overly depth and close to anaesthetic stage IV (Haskins, 2015). The four phases of stage III have not been studied in decapods yet. Notable, signs of anaesthetic state can be similar to death, thus anaesthetic overdose may cause animal mort (O'Brien, 2008). For this reason, anaesthesia depth should be as low as possible (Dombrowski & De Voe, 2007). If unsure how depth the anaesthesia is, the practitioner should decrease the depth and adjust to the appropriate level. Fig. 1 illustrates the general anaesthesia triad, while Fig. 2 illustrates the anaesthetic state in decapod crustaceans, divided into the four stages of anaesthesia.

Anaesthetic depth depends on its purpose, e.g., animal transportation may require animal sedation (stage I), while invasive procedures require surgical anaesthesia (stage III). For procedures of short duration, anaesthesia achieved during induction time may be sufficient to ensure an adequate anaesthetic level and conclude the procedure. For long-duration ones, maintenance of the anaesthetic state is required. This can be achieved by reapplying the chosen anaesthetic agent or using an anaesthetic of long-term effect. When available, balanced anaesthesia is recommended. As no single anaesthetic agent perfectly achieves all anaesthetic effects, by simultaneously combining two different anaesthetic agents, their anaesthetic effects are associated, e.g., anaesthesia potency, fast recovery, and better control of each anaesthetic effect (Wouden & Miller, 2012). Moreover, by mixing different anaesthetic agents, their final formulation may be more suitable for some uses. For example, eugenol is not easily soluble in water but, when mixed with menthol plus ethanol as solvent, the blend remains suspended in the water column, besides being efficient to impair anaesthesia in prawns by acting in different receptors (Saydmohammed & Pal, 2009).

### 4.2 Induction and routes

There are two mains common vias decapod crustaceans can be anaesthetised, i.e., parentally and via immersion. Alternatively, volatile agents, local agents, and alternative vias are also possible.

**Fig. 3:**
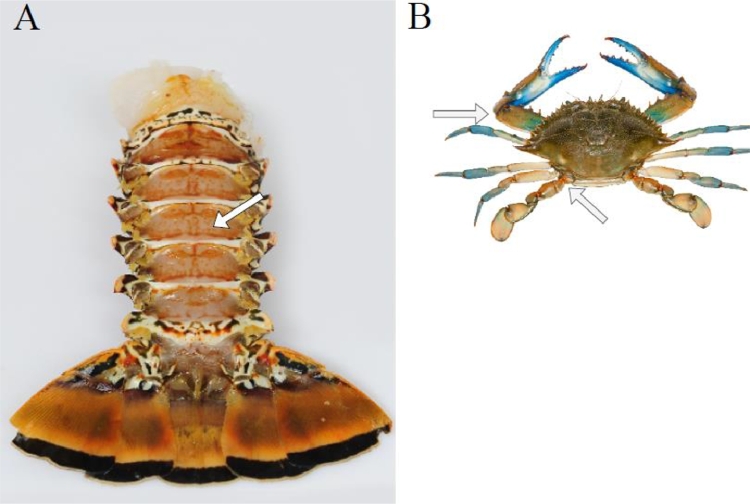
Arthrodial membrane and potential sites of injection indicated by arrows. Lobster, tail ventral view, between the abdominal joins segments **(A)**. Crab, dorsal view, between the segments of the chela & between the cephalothorax and the swimming leg's coxa **(B)**.

• **Parentally**: injection with injectable anaesthetic into the haemocele via the arthrodial membrane (Fig. 3, Table 2). Also called intersegmental membrane, the arthrodial membrane is a soft, noncalcified cuticle where the exoskeleton becomes thin, particularly on the joints of appendages and between the ventral intersegmental structure (Bitsch & Bitsch, 2002; Williams et al., 2003). As this reaches haemolymph, it is also called intravascular via (Quesada et al., 2011). Insulin syringe and corresponding needle are commonly used.

**Table 2:** Efficacy of injectable anaesthetic agents for decapod crustaceans.

Anaesthetic	Species	Dose	Induction time (min)	Anaesthetic effect	Anaesthesia duration (min)	Notes	Refs.
**Crabs**							
Alphaxalone	*Callinectes sapidus* Rathbun, 1896	15 mg kg^−1^ 100 mg kg^−1^	0.3 0.4	Sedation Anaesthesia	11.3 66.1	High dose: bradycardia, limb autotomy followed by death. IV	Minter et al. 2013
Eugenol	*Carcinus maenas* Linnaeus, 1758	0.15 µl g^−1^	13 – 17	N/C	62 – 72	Pericardial sac injection	Huntsberger, 2012
Ketamine-xylazine	*Callinectes sapidus* Rathbun, 1896	20:20 mg kg^−1^	0.5 – 0.6	Short term	5 – 10	Bradycardia; IV	Quesada et al. 2011
Ketamine-HCl	*Pseudocarcinus gigas* Lamarck, 1818	0.025 mg kg^−1^ 0.05 mg kg^−1^ 0.1 mg kg^−1^	0.2 – 0.7	Short – medium term	15 25 40	Cheliped rigidity; excitatory phenomena; IV	Gardner, 1997
Morphine-HCl	*Neohelice granulata* Dana, 1851	50 µg g^−1^ 100 µg g^−1^ 150 µg g^−1^	6 ^†^ 18.8 ^†^ 27 ^†^	Short term (when compared to vertebrates)	45 61.4 69.8	Cephalothorax-abdominal membrane injection	Lozada et al. 1988
Nano encapsulated *Melaleuca alternifolia* Cheel,1924 EO	*Neohelice granulata* Dana, 1851	40 µl	8	Short term	9	Undiluted; Arthrodial membrane injection	Souza et al. 2018
Procaine	*Carcinus maenas* Linnaeus, 1758 *Cancer paguros* Linnaeus, 1758	25 mg kg^−1^	0.3 – 0.5	Long term	120 - 180	Excitatory phenomena (10 sec); IV	Oswald, 1977
Terpinen-4-ol (major compound of *Melaleuca alternifolia* Cheel,1924 EO)	*Neohelice granulata* Dana, 1851	20 µl	39	Medium term	30	Undiluted; Arthrodial membrane injection	Souza et al. 2018
Tiletamine-zolazepam	*Callinectes sapidus* Rathbun, 1896	30 mg kg^−1^	0.3 – 1.0	Short term	5 – 7	Bradycardia; IV	Quesada et al. 2011
Xylazine	*Carcinus maenas* Linnaeus, 1758 *Cancer paguros* Linnaeus, 1758	70 mg kg^−1^	5 – 6	Medium term	45	IV	Oswald, 1977
Xylazine	*Pseudocarcinus gigas* Lamarck, 1818	16 mg kg^−1^ 20 mg kg^−1^	3 – 5	Short – medium term	25 45	IV	Gardner, 1997
**Hermit crab**							
Xylazine	*Coenobita clypeatus* Fabricius, 1787	70 mg kg^−1^	N/C	Short term	5 – 10 300 - 360 (full recovery)	Intrabdominal injection	McMahon & Burggren, 1979
**Lobster**							
Eugenol	*Homarus americanus* Edwards, 1837	0.15 µl.g^−1^	8 – 15 10	18°C 12°C	65 – 75 20 – 30 min	Pericardial sac injection; Eventual paralysis of leg or tail due to damage to adjacent muscle	Huntsberger, 2012
Isobutanol 100%	*Homarus americanus* Edwards, 1837	2.0 µl.10 g^−1^ 4.0 µl.10 g^−1^	2 1	Medium to long term	19 184	Excitatory phenomena (0.5 – 2 min); water temperature: 6.0 – 8.5°C; injection into the abdominal sinus	Gilgan, Burns 1976
**Crayfish**							
Ketamine	*Orconectes virilis* Hagen, 1870	90 µg g^−1^	1	Deep anaesthetization; long term	110	IM injection on the tail	Brown et al. 1996
Lidocaine	*Orconectes virilis* Hagen, 1870	400 µg g^−1^	1.5	Light anaesthetization; short term	25	IM injection on the tail	Brown et al. 1996

Sec: seconds. Min: minutes. H: hour. N/C: not commented. IV: intravascular injection. IM: intramuscular injection. EO: essential oil. ^†^: response observed in 50% of the animals.
• **Immersion**: bath in an aqueous anaesthetic agent (Table 3), which is absorbed mainly via gills; these agents should be slowly added into the water until the animal reaches the desirable anaesthetic stage (Zachariah & Mitchell, 2009).

**Table 3:** Efficacy of aqueous anaesthetic agents and halothane for decapod crustaceans.

Anaesthetic	Species	Dose	Induction time (min)	Anaesthetic effect	Anaesthesia duration (min)	Notes	Refs.
**Crabs**							
Clove oil	*Cancer magister* Dana, 1852	1.0 ml l^−1^	< 30	Smooth	10	Ethanol as solvent (1:9)	Morgan et al., 2001
Clove oil	*Hemigrapsus oregonensis* Dana, 1851	3.0 ml l^−1^	< 90	Smooth	65	Ethanol as solvent (1:9)	Morgan et al., 2001
Clove oil	*Portunus sanguinolentus* Dana, 1851	0.15 ml l^−1^ 0.20 ml l^−1^ 0. 25 ml l^−1^	18 – 22 12 – 14 5	Smooth	24 – 29 34 – 49 92 - 108	Absolute ethanol as solvent	Premarathna et al., 2016
Clove oil	*Pseudocarcinus gigas* Lamarck, 1818	0.125 ml l^−1^	30	Smooth	Short	Ethanol as solvent	Gardner, 1997
Clove oil	*Pugettia producta* Randall, 1840	0.06 ml l^−1^	< 5	Smooth	14	Ethanol as solvent (1:9)	Morgan et al., 2001
**Hermit crabs**							
Clove oil	*Coenobita clypeatus* Fabricius, 1787	Watery solution	N/C	Sedation	N/C	For examination and measurement	Sanvicente-Añobe & Hermoso-Salazar, 2011
Lidocaine	*Coenobita compressus* Edwards, 1837	Watery solution	1 – 3	Sedation	N/C	N/C	Osorno et al., 1998
**Lobsters**							
AQUI-S® (Isoeugenol 50%)	*Jasus edwardsii* Hutton, 1875	40 mg l^−1^	10 – 20	Sedation	Short	Foam produced by lobster	Robertson et al., 2018 AQUI-S® (n.d.)
		200 mg l^−1^		Anaesthesia			
AQUI-S® (Isoeugenol 50%)	*Jasus edwardsii* Hutton, 1875	80 mg l^−1^	30	N/C	N/C	Criteria: loss of equilibrium	Forgan et al., 2014
Eugenol 99%	*Nephrops norvegicus* Linnaeus, 1758	600 µl l^−1^	5 – 9 ^†^	Short term	6 – 16 ^†^	Absolute ethanol as solvent (1:9)	Cowing et al., 2015
		900 µl l^−1^	3 – 5 ^†^		6 – 18 ^†^		
Isobutanol	*Homarus americanus* Edwards, 1837	1.5 ml l^−1^ 1.8 ml l^−1^ 3.6 ml l^−1^	9 – 15 5 – 11 1 – 3	Sedation	9 – 40 12 – 45 10 – 20	Excitatory phenomena; rigid body	Foley et al., 1966
**Crayfish**							
Clove oil	*Cherax quadricarinatus* von Martens, 1868	500 µl l^−1^	12 – 13 ^†^	Short term	8 – 9 ^†^	Ethanol 96% as solvent (1:6)	Ghanawi et al., 2019
Isobutanol	*Panulirus homarus* Linnaeus, 1758 *Panulirus ornatos* Fabricius, 1798 *Panulirus polyphagus* Herbst, 1793 *Panulirus versicolor* Latreille, 1804	50 ppm	25	N/C	N/A	Water temperature: 28°C; Recommended for transportation	Pozhoth & Jeffs, 2022
**Shrimp**							
*Aloysia triphylla* Britton,1925 EO	*Macrobrachium rosenbergii* de Man, 1879	225 µl l^−1^ 300 µl l^−1^	5 ^†^ 5 ^†^	Short term	10 ^†^ 9 ^†^	Ethanol as solvent (1:10); Water hardness 68 mg.l^−1^	Cagol, 2020
*Aloysia triphylla* Britton, 1925 EO	*Penaeus vannamei* Boone, 1931 (SA)	300 µl l^−1^	< 8.3 ^†^	Short term	< 16.6 ^†^	Ethanol as solvent (1:10)	Parodi et al., 2012
*Aloysia triphylla* Britton, 1925 EO	*Penaeus vannamei* Boone, 1931 (PL)	300 µl l^−1^	∼ 10 ^†^	Short term	∼ 10 ^†^	Ethanol as solvent (1:10)	Parodi et al., 2012
*Cymbopogon citratus* (DC) Stapf, 1906 EO	*Penaeus vannamei* Boone, 1931	10 µl l^−1^	Initial stages of exposure	Light sedation	Partial: 120 Total: 240	Ethanol as solvent (1:10)	Becker et al., 2021
Eugenol	*Fenneropenaeus indicus* Edwards, 1837	1.3 mg l^−1^ (PL)	N/C	Sedation	240	Recommended for transportation	Akbari et al., 2010
Eugenol (80%)	*Penaeus semisulcatus* De Haan, 1844	100 mg l^−1^ 150 mg l^−1^	4.5 - 5.5	Short term	9.5 - 12.5 2.6 - 3.4	Recommended water parameters: temperature 25 – 35°C Salinity 25 - 40 ppt DO: > 6 mg.l^−1^	Soltani et al., 2004
Eugenol (99%)	*Penaeus vannamei* Boone, 1931 (SA)	200 µl l^−1^	< 8.3 ^†^	Short term	∼ 8.3 ^†^	Ethanol as solvent (1:10)	Parodi et al., 2012
Eugenol (99%)	*Penaeus vannamei* Boone, 1931 (PL)	175 µl l^−1^	< 3.3 ^†^	Short term	∼ 3.3 ^†^	Ethanol as solvent (1:10)	Parodi et al., 2012
Halothane	*Penaeus vannamei* Boone, 1931	0.5 mg l^−1^ 1.0 mg l^−1^ 1.5 mg l^−1^ 2.0 mg l^−1^ 2.5 mg l^−1^	6.5 5 4 3 2.5	Short term	11 12 14 15 17	Short procedures	Guzmán-Sáenz et al., 2010
Lidocaine	*Penaeus vannamei* Boone, 1931	400 mg l^−1^ 500 mg l^−1^ 600 mg l^−1^ 700 mg l^−1^ 800 mg l^−1^	5.5 5 4 3.5 2.5	Short – medium term	18 21 22 22 24	Medium term recovery	Guzmán-Sáenz et al., 2010
*Lippia alba* (Miller) Brown, 1925 EO	*Penaeus vannamei* Boone, 1931 (SA)	750 µl l^−1^	< 16.6 ^†^	Short term	∼ 8.3 ^†^	Ethanol as solvent (1:10)	Parodi et al., 2012
*Lippia alba* (Miller) Brown, 1925 EO	*Penaeus vannamei* Boone, 1931 (PL)	500 µl l^−1^	∼ 10 ^†^	Short term	∼ 10 ^†^	Ethanol as solvent (1:10)	Parodi et al., 2012
*Santalum* sp. Linnaeus, 1753 EO	*Penaeus schmitti* Burkenroad, 1936	0.5 ml l ^-1^	< 5 ^†^	Sedation	< 16 ^†^	Recommended for transportation; Ethanol as solvent (1:9)	Matulovic & Oshiro, 2016
**Prawn**							
AQUI-S® (Isoeugenol 50%)	*Macrobrachium rosenbergii* de Man, 1879	100 mg l^−1^	30	Sedation	16 – 40	Compound added to water 5 min before animals	Coyle et al., 2005
Clove oil (eugenol)	*Macrobrachium rosenbergii* de Man, 1879	100 mg l^−1^	15	Sedation	15 – 21	Compound added to water 5 min before animals	Coyle et al., 2005
Eugenol	*Macrobrachium rosenbergii* de Man, 1879	200 µl l^−1^	30 - 32	Mild anaesthesia	15 – 19	Ethanol as solvent	Saydmohammed & Pal, 2009
Eugenol + menthol	*Macrobrachium rosenbergii* de Man, 1879	200 µl l^−1^	27 - 30	Mild anaesthesia	17 – 20	Ethanol as solvent; Balanced anaesthesia	Saydmohammed & Pal, 2009
Clove oil (95% eugenol)	*Macrobrachium rosenbergii* de Man, 1879 (PL)	5 drops in 5 ml ethanol into 1000 ml water	4 min	Short term	N/C	Increased ROC lasting 1 h after recovery	Taylor et al., 2002
Clove oil (82 - 87% eugenol)	*Macrobrachium tenellum* Smith, 1871	300 µl l^−1^ 600 µl l^−1^ 900 µl l^−1^	12 – 23 8 – 16 5 -11	Short – mild term	12 -26 24 – 30 18 - 50	Recommended for transportation and handling	Aréchiga-Palomera et al., 2016
Eugenol	*Penaeus monodon* Fabricius, 1798	100 mg l^−1^	8	N/C	13	95% ethanol as solvent (1:9)	Jiang et al., 2020

Sec: seconds. Min: minutes. H: hour. N/C: not commented. PL: post larvae. SA: sub-adult. ROC: resting oxygen consumption. DO: dissolved oxygen. EO: essential oil. ^†^Originally presented as seconds, original values divided by 60.
• **Inhalation anaesthetic agents**: terrestrial and facultative air breathers should be placed on an appropriate anaesthetic chamber. For aquatic species, gaseous agents can be administered with oxygen, being both pumped into the water; alternatively, vapour agents, that is liquid at ambient temperature and pressure, can be mixed in the water. Potential options include halothane, enflurane, and isoflurane (Table 4, Borozins et al., 2020; Guzmán-Sáenz et al., 2010; Obradović, 1986). The quantity of inhalation agents can be presented in three ways: pressure (e.g., mmHg), concentration (e.g., vol.%), and mass (e.g., mg). The most used is concentration, where the percentage of a certain agent is given in relation to the total volume of the gas mixture (Steffey et al., 2015).

**Table 4:** Efficacy of inhalation anaesthetic agents for decapod crustaceans.

Anaesthetic	Species	Dose	Induction time (min)	Anaesthetic effect	Anaesthesia duration (min)	Notes	Refs.
**Crayfish**							
Halothane	*Astacus astacus* Linnaeus, 1758	0.5 vol.%	< 25	Long term	<150	Air-breathing acclimation for 30 min; cotton wool soaked on halothane and let it dry inside glass bell; stimulation of muscle tonus with limb and tail contortion.	Obradović, 1986
• **Local anaesthetic agents**: locally applied to aim at desensitisation of a specific part of the body, preserving the consciousness of the animal (Table 5). Some local agents may be used systematically, e.g., lidocaine (Garcia, 2015).

**Table 5:** Efficacy of local anaesthetic agents for decapod crustaceans.

Anaesthetic	Species	Dose	Local of application	Method of application	Notes	Refs.
**Shrimp**						
Lidocaine	*Penaeus vannamei* Boone, 1931	2.5%	Eyestalk	N/C	Normal feeding; 6% erratic swimming	Taylor et al. 2004
**Prawn**						
Benzocaine	*Palaemon elegans* Rathke, 1836	2%	Antenna	Topic with small brush	Aversive short reaction (tail flicking scape response, antenna overgrooming)	Barr et al. 2008

N/C: not commented.
• Alternative routes: **intracardiac injection** (Quesada et al., 2011).

The choice of the route depends on equipment and anaesthetic availability, the expertise of the anaesthetist, and the size of the animal. Small animals such as little shrimps may be difficult to receive injectable agents, with high chances of overdose and iatrogenic damage (O'Brien, 2008). In general, the small the animal, the high is the dose per bodyweight of the anaesthetic (Flecknell, 2008). The necessary volume to be injected thus may be a limiting factor; a proportionally large volume of anaesthetic to the bodyweight will be detrimental and painful. Many intravascular agents rapidly induce anaesthesia, typically in seconds (e.g., alphaxalone, ketamine, ketamine-xylazine, tiletamine-zolazepam). Notable, intravascular agents, after being injected, cannot be removed from the animal body but metabolically. Injection with parenteral agents should ideally start with a half dose injection followed by a slow injection of the remaining dose until reaching the desirable anaesthetic depth (Flecknell, 2008). Anaesthetists must be careful and obviate severe depression of the nervous system, which is difficult to revert and may result in ventilatory failure, cardiac arrest, and animal death.

The choice of the anaesthetic agents depends on the animal species, animal weight, availability of equipment, practitioner skills, and the procedure requirements which determine the necessary anaesthesia duration and deep. Severely noxious stimuli (e.g., invasive procedures with exposure to internal organs) require higher anaesthetic pressure (i.e., higher level of anaesthetic and deep) than less noxious stimuli (e.g., mouth swab for sampling) or immobilization for animal examination. Similarly, anaesthetic dose and exposure time, environmental factors, and animal body weight, gender, and physiological condition directly influence the anaesthetic effectiveness. For example, ovigerous females or wild animals may present a longer induction time and a faster recovery time than non-ovigerous females or animals previously kept in captivity (Cowing et al., 2015). Noteworthy, the anaesthetic agent and its dose varies with the animal species, being particular to each case. Being a given anaesthetic and its dose effective and safe for a certain decapod species is not a guarantee this given anaesthetic and dose will necessarily be effective and safe for another species of decapod, even among similar groups, such as shrimps and prawns, or lobsters and crayfish, or crabs and hermit crabs. The practitioner should attentively study the case of the animal and the best choice available for that species.

Similarly, the induction and recovery times increase with the animal body weight and aerial exposure time (Ghanawi et al., 2019; Jiang et al., 2020; Li et al., 2018). The smaller the animal's body weight, the higher is the oxygen consumption and, consequently, the higher is the anaesthetic concentration per time into the body and, thus, the shorter is the induction time (Jiang et al., 2020). Also, the induction time decreases with high water temperature and high anaesthetic concentration (Li et al., 2018). Intertidal decapods (e.g., H. oregonensis Dana, 1851), due to their capacity of breath-holding and anaerobic metabolism, may present higher induction time on immersion anaesthesia than primarily subtidal animals (e.g., Cancer magister Dana, 1852) which, in turn, may present higher induction time than typically subtidal decapods (e.g., Pugettia producta Randall, 1840, Morgan et al., 2001). Furthermore, season influences anaesthetic dose on lobster. Huntsberger (2012) demonstrated that a higher anaesthetic dose may be required to achieve an anaesthetic state in *Homarus americanus* Edwards, 1837 during spring-early summer than during end summer-autumn. That is, a dose considered adequate for *H. americanus* Edwards, 1837 in high temperatures can result in animal death if used in low temperatures. The author theorizes the high lipid content of the gonads present during spring-summer time absorbs the anaesthetic that would be surplus and detrimental during end summer-autumn time.

### 4.3 Synthetic anaesthetic agents for decapods

**Alphaxalone** is a neuroactive steroid, i.e., a steroid that affects neuronal excitability through the modulation of GABA and glutamate receptors (Forman et al., 2012, Martinez-Botella et al., 2014). Through the activation of GABA receptors, alphaxalone induces anaesthesia (Forman et al., 2012; Minter et al., 2013). **Isobutanol**, also called isobutyl alcohol, was described as a sedative and anaesthetic for lobsters and crayfish.

Its mechanism of action was not found in the literature. Adverse effects reported in decapods include excitatory phenomena and rigid body (Foley et al., 1966; Gilgan & Burns, 1976). Isobutanol, when added to the water in a tentative anaesthetic bath, may lead to erratic anaesthesia (Gilgan & Burns, 1976).

**Morphine** is an opioid and acts on the opiate receptors via blocking the presynaptic release of neurotransmitters and diminishing the postsynaptic response to excitatory neurotransmitters (Griffin & Woolf, 2012; Lozada et al., 1988). Notable, morphine may induce behaviour sensitization in decapods (Nathaniel et al., 2010). This phenomenon occurs when a given drug intensifies the motor stimulant response, commonly a progressive and long-term effect associated with the repeated exposure to that given drug (Steketee & Kalivas, 2011). Crayfish that received an intra-pericardial infusion of morphine presented stereotypic behaviour, including grooming, tail flipping, mouthpart movements, repetitive actions, and mild tremor (Nathaniel et al., 2010). In comparison to the morphine response of vertebrates, arthropods such as decapods have a much faster time of metabolization, i.e., decapods present a short anaesthesia time than vertebrates when anaesthetized with morphine. This can be attributed to the greater vascular permeability of the arthropods' brain barrier compared to the one on vertebrates (Lozada et al., 1988).

**Xylazine** is an α_2_-adrenergic receptor agonist and its antinociception effect is described on vertebrates and involves the activation of presynaptic α_2_ receptors and synaptic inhibition on the central nervous system (Wixson, 1994). Receptors for α_2_ agonists are found both pre and post-synaptically (Lorens, 2011). Xylazine can be administered in association with other sedatives and anaesthetics to potentiate their effect, e.g., ketamine-xylazine (Rakin, 2015; Wixson, 1994). Adverse effects on vertebrates include significant bradycardia, cardiac arrhythmia, bradypnea, salivation, emesis, and reflux (Rankin, 2015; Wixson, 1994). In decapods, Quesada et al. (2011) observed bradycardia on crabs after intravascular administration of ketamine-xylazine.

From the cyclohemaxines class, **ketamine** and **tiletamine** are dissociative anaesthetics, thus leading to a cataleptic state. They prevent the binding of glutamate receptors and have action on opioid receptors (Berry, 2015; Caulkett & Arnemo, 2015). Tiletamine is only available in combination with **zolazepam**, a benzodiazepine agonist (Caulkett & Arnemo, 2015). Dissociative anaesthetics do not interact with the GABA receptors (Berry, 2015). Cyclohemaxines, if administered alone, may cause muscle hypertonus and twitch in nonhuman vertebrates (Caulkett & Arnemo, 2015), while ketamine has psychomimetic effects in humans (Griffin & Woolf, 2012). In decapod, described side effects include excitatory phenomena, bradycardia, and cheliped rigidity (Gardner, 1997; Quesada et al., 2011). Balanced anaesthesia, particularly ketamine co-administered with a sedative, is recommended (Caulkett & Arnemo, 2015).

Among the earliest inhalation anaesthetics, **halothane** is a hydrocarbon with high anaesthetic potency, though commonly producing slow induction and recovery (Wouden & Miller, 2012). Inhalation anaesthetics affect the central nervous system, however, their precise mechanism and site of action, or where the nociceptive transmission is interrupted remain unknown (Steffey et al., 2015). Despite halothane being usually administered inhaled, it was also described in immersion anaesthesia for shrimps (Guzmán-Sáenz et al., 2010). Reported adverse effects include cardiac arrhythmias in vertebrates (Steffey et al., 2015), and vomiting along with tail and limb contortions as a result of strong muscle tonus stimulation in decapods (Obradović, 1986).

**Benzocaine, lidocaine,** and **procaine** are local anaesthetics. Benzocaine and procaine are amino-esters, while lidocaine is amino-amides (Garcia, 2015). Local anaesthetics inhibit the action potential propagation by blocking the sodium channel, thus suppressing the nociceptive transmission (Barr et al., 2008; McKune et al., 2015; Schulman & Strichartz, 2012). They are moderately hydrophobics, as they diffuse on and dissociate from the cellular membrane into the cytoplasm (Schulman & Strichartz, 2012). Some can be administered systematically, e.g., lidocaine and procaine. Notable, benzocaine may cause methemoglobinemia in several vertebrate species (Garcia, 2015), although a possible side effect on decapod hemocyanin is unknown. Reported adverse effects on decapods include excitatory phenomena, erratic swimming, scape response, and overgrooming (Barr et al., 2008; Brown et al., 1996; Oswald, 1977; Taylor et al., 2004).

### 4.4 Natural compounds-based anaesthetics

The use of natural products is notably increasing, and many studies have been conducted on the development and establishment of herbal medicine for veterinary purposes. **Eugenol** and **isoeugenol** are among the most used and effective herbal anaesthetics for decapod crustaceans, being absorbed mainly by the gills. Eugenol (4-allyl-2-methoxyphenol) and isoeugenol (2-methoxy-4-propenylphenol) are phenylpropenes naturally produced by leaves and stem of plants such as clove and allspice to attract pollinators but are also antimicrobial and anti-animal plant protective compounds (Koeduka et al., 2006, PubChem, 2021). Isoeugenol can also be synthetically obtained via eugenol isomerization (Červený et al., 1987). In commercial, pure preparations, 1 ml of clove oil contains 1 g of eugenol; for a solution of 100 mg ml^−1^ of clove oil, dilute one part of clove oil in nine parts of 95% ethanol (Lewbart, 2018). Eugenol appears to be feasible for ovigerous female species, without detrimental effect on the embryo's development (Gardner, 1997). Eugenol is hydrophobic, thus a solvent (e.g., ethanol or polysorbate – the last used in commercial preparations) should be used (Lewbart & Yanong, 2018), commonly 1 part of clove oil to 9 parts of ethanol. Due to eugenol volatility, it penetrates the animal body and is excreted faster in warm temperatures, thus its effect and duration are dependent on the environmental temperature (Wycoff et al., 2018). What is regarded eugenol mode of action, it is dose-dependent and reversibly reduces the neural activity by blocking the sodium, calcium, and potassium channels, causing decreased activity on proprioceptors (Cowing et al., 2015; Wycoff et al., 2018). AQUI-S® and AQUI-S® 20E (AQUI-S New Zealand Ltd., Lower Hutt, New Zealand) are commercial formulations containing 50% isoeugenol and 10% eugenol, respectively; they are recommended, by the manufacturer, for fishes, abalone, and aquatic crustaceans. Also, FA-100 (DS Pharma Animal Health, Osaka, Japan) is a eugenol commercial formulation recommended for the anaesthetic bath for crustaceans and fishes (Miyaki et al., 2020, DSPAH personal communication). Balanced anaesthesia can be achieved by combining eugenol and menthol, while menthol is ineffective as a single anaesthetic agent (Saydmohammed & Pal, 2009). Prolonged exposure to eugenol (e.g., keeping the animal for a long term in the same anaesthesia bath with excreted eugenol) may lead to depression to suppression of ventilation and cardiac function (Wycoff et al., 2018). Eugenol is deleterious to sessile marine invertebrates and corals (Boyer et al., 2009; Calado, 2012), thus should not be used in aquariums with those animals.

Several natural compounds-based anaesthetics for decapod crustaceans are essential oils. Essential oils are a blend of volatile compounds physically extracted from plants, part of it or the whole plant (Franz & Novak, 2020). They are composed of different compounds in different concentrations, and their sedative and anaesthetic effects can be due to a main active compound, a synergic effect of the active compounds, or a combination of their different phytoconstituents (Aydin & Barbas, 2020; Franz & Novak, 2020). For instance, the major compounds of ***Aloysia triphylla*** (Britton, 1925; c.n. lemon verbena) include α-citral, limonene, and β-citral (Sgarbossa et al., 2019); and of ***Lippia alba*** ((Miller) Brown, 1925; c.n. lemon balm), citral, linalool, β-caryophyllene, carvone, and tagetenone (Hennebelle et al., 2008). While ***Cymbopogon citratus*** ((DC) Stapf, 1906; c.n. lemongrass) is rich in citral, geraniol, and linalool (Hacke et al., 2020), ***Santalum* sp**. (Linnaeus, 1753; c.n. sandalwood) in α-, β- and epi- β-santalols (Silva et al., 2018), and ***Melaleuca alternifolia*** (Cheel, 1924; c.n. tea tree) is rich in terpinen-4-ol, terpinene, cineole, and terpinolene (Hart et al., 2000; Homer et al., 2000). The sedative and anaesthetic effects of essential oils are believed to be related to opioid and glutamate receptors, and the GABAergic system, an inhibitory neurotransmitter system (Becker et al., 2021; Rial et al., 2014; Tsuchiya, 2017). Noteworthy, natural compounds should not be automatically assumed as safe because they are natural, but their use should be based on formal research. Similarly, the anaesthetist should keep records of the use of natural compounds-based anaesthetic and consider any previous use of herbal medicines on the anaesthetic plan.

### 4.5 Discouraged procedures and chemicals

Some procedures and chemicals are known to provide inadequate analgesia, or their analgesic and anaesthetic effects are questionable in decapod crustaceans. While it is uncertain whether they are appropriate or not, it is preferable to grant the benefit of the doubt than to cause discomfort or pain to the animal. For example, **hypothermia** does not provide analgesia, but only immobilization and torpor, that is, even if the animal is immobile, sensation and responses to painful stimuli are still present (Birch et al., 2021; Gressler et al., 2021; Pellet et al., 2020). Hypothermia should never be used or be acceptable for any painful or invasive procedure (Cooper, 2004). Cooling may also result in autotomy of appendages (O'Brien, 2008; Oswald, 1977). Similarly, the use of **carbon dioxide** (CO_2_) is discouraged. It is uncertain if CO_2_ provides adequate analgesia or only immobilize the animal (Cooper, 2004; O'Brien, 2008) and, as CO_2_ lows water pH, decapod crustaceans show signs of aversion and discomfort when exposed to CO_2_ (Fregin & Bickmeyer, 2016).

**Benzocaine** (except as topic anaesthetic)**, chlorpromazine, chloroform, dexamethonium bromide, d-tubocurarine, ethanol, ether, etomidate, gallamine triethiodide, guaiacol glyceryl ether, lignocaine, magnesium chloride and magnesium salts, quinaldine, suxamethonium chloride, tubocurarine**, and **2-phenoxyethanol** are ineffective or detrimental to decapod crustaceans (Foley et al., 1966; Jensen et al., 2013; O'Brien, 2008; Oswald, 1977; Quesada et al., 2011; (Fregin & Bickmeyer, 2016)). **Chlorpromazine hydrochloride** causes immediate excitement followed by autotomy of appendages (Oswald, 1977). **Ethanol**, besides being described as ineffective in most studies, can lead to agitation and disorientation (Becker et al., 2021). **Mint and lavender essential oils** are irritant agents and shrimps exposed to them show signs of pain and discomfort; additionally, the lavender essential oil may increase shrimps’ haemolymph glucose, indicating physiological stress (Matulovic & Oshiro, 2015). **Passionflower extract** (*Passiflora incarnata* Linnaeus, 1753) and **valerian** (*Valeriana officinalis* Linnaeus, 1753) are ineffective as anaesthetics to the prawn *Macrobrachium tenellum* Smith, 1871 (Aréchiga-Palomera et al., 2016). **Intracardiac ketamine** causes cardiorespiratory arrest and animal death or prolonged recovery; pentobarbital induces dysphoria and violent excitement; propofol causes contractions, tremors, autotomy, and seems to induce pain (Quesada et al., 2011). **Xylazine**, despite being effective on some decapods, causes partial sedation and unstable induction in blood-spotted crabs (e.g., *Portunus sanguilonentus* Herbst, 1783), thus it is inadvisable to be used in these animals (Premarathna et al., 2016). **Tricaine methanesulfonate** (MS-222), used as an injectable agent, volatile agent, or by immersion, is ineffective to produce anaesthesia and may result in autotomy (Mosley & Lewbart, 2014; O'Brien, 2008; Obradović, 1986; Oswald, 1977).

## 5 Post anaesthetic care and attention

Postoperative or post-procedure analgesia and care reduce animal pain and discomfort, thus reducing recovering time and guarantying animal welfare. Notable, analgesic agents better work when administered before the pain stimulus is perceived (Wolfensohn & Lloyd, 2013). Restore of normal motility, including movements of the limb, abdominal flaps, and chelae, associated with the reestablishment of defence behaviour and righting reflex indicate recovery of anaesthesia (Mosley & Lewbart, 2014). For anaesthesia recovery, the animal should receive medical-grade oxygen or pure air, or clean dechlorinated water (Zachariah & Mitchell, 2009). Animals under recovery must be placed in a separated tank/environment, isolated from unanaesthetised animals, with minimal handling and disturbance, in individual clean compartments ideally in a separated recovery room. Anaesthetized or sedated animals should never be placed together with unanaesthetised animals, as there is a risk of the active one attack and injury the passive one (Noga et al., 2020; Oswald, 1977). The temperature should be kept constant, with minimal environmental disturbance, high water quality with aeration for aquatic species, ideal relative air humidity for hermit crabs (70%–90%), and darkened environment when possible (Judah & Nuttall, 2008; Wolfensohn & Lloyd, 2013). In warm temperatures or tropical places, anaesthetic recovery may occur faster than in cold weather, as warm conditions better support ectothermic animals (Cooper, 2011). Similarly, live food should not be offered for an anaesthetized or sedated animal, as they will probably not eat promptly after anaesthesia and may be attacked by the live invertebrates offered (O'Brien, 2008). Only hydrate and fully recovered animals should be fed. The animal should be monitored and receive supportive care until fully recovery; only after proper examination and complete recovery animal can return to the original tank/terrarium.

Fluid balance management is not critical in aquatic animals, though it should be monitored in aquatic animals when they are kept out of the water and in terrestrial animals or those with natural air-exposure behaviour. Attention to haemolymph loss and penetrating wounds or surgery conditions per where fluids and electrolytes can enter or leave the body (Cooper, 2011; Pellett & O'Brien 2019). Haemolymph loss higher than 1% of the animal body weight can be lethal in minutes (Cooper, 1998). For terrestrial species, fluid therapy can be oral, offering an open source of clean, dechlorinated water or saline, such as a shallow container to prevent the drowning of the animal; one may also suggest the use of cotton soaked in water or saline, although this can result in unhygienic conditions and might prevent the proper access to the water on the dish (Zachariah, 2020). For aquatic species, fluid therapy can be via parenteral injection of rehydration solution (e.g., saline solution) on the arthrodial membrane (Cooper, 1998; Marnell, 2016; Zachariah, 2020). If an animal takes a long time to recover or seems to be dead, medical-grade oxygen should be pumped into the water or holding tank for ∼ 20 min, continually, or for one to two hours, periodically (Cooper, 2011). As death in decapod is difficult to infer, it is advisable that only after 24 h of immobility, absence of eye-withdraw reflex, and flaccid body the decapod should be inferred to be truly dead (Gilgan & Burns, 1976). Dombrowski & De Voe (2007) recommend assuming an invertebrate animal is dead only after postmortem rigidity or signs of body deterioration.

## 6 Final considerations

All animals have the right to be protected from pain, discomfort, suffering, or stress. Over the last decades, we observed a great advance in the knowledge of decapod crustaceans’ anatomy, physiology, veterinary care, and behaviour. Nevertheless, their welfare, protection, and veterinary assistance are still neglected and undervalued, when compared with vertebrates’ animals. As concern their anaesthesia, there is a largely unknown and a vast area to be improved. Among the areas that lack research and development are the clinical monitoring of the different anaesthetic stages and depth; advancement on pain recognition and control; improvement of the current anaesthesia protocols; balanced anaesthesia; new anaesthetics; pharmacodynamics, pharmacokinetic, and drug interaction; improvements on veterinarians and other practitioners' skills and expertise; advancements on the legal requirement to ensure pain-free procedures, protecting the rights of decapod crustaceans to be free from distress, discomfort, suffering, and pain. Likewise, all and any suggested anaesthetic agent and procedure should be continuously evaluated and refined and, if necessary, replaced or discontinued. Contemporary veterinary medicine and related areas are facing increasing demand for invertebrate care, requiring new and sophisticated techniques along with specialized knowledge. Invertebrates, including decapods, must receive veterinary and ethical treatment, including the best of our skills and available tools to ensure their right to be free of pain is safeguarded by all veterinarians and other practitioners when dealing with them.

## Funding

This review did not receive funding from any source.

## Ethical statement

This review does not contain any study or trial with human participants, non-human vertebrates, or invertebrates’ animals performed by the author. No specific authorization is required.

## Declaration of Competing Interest

The authors declare that they have no known competing financial interests or personal relationships that could have appeared to influence the work reported in this paper.

## Appendix Supplementary materials

Image, application 1

Image, application 2
