# Supplementary material for: Anaesthesia of decapod crustaceans
Source: Vet Anim Sci. 2022 May 14;16:100252. doi: 10.1016/j.vas.2022.100252 (PMC9127210; doi:10.1016/j.vas.2022.100252)
Supplement: Supplementary file 1 [file mmc1.pdf]

Record № ______________ Date: __/__/__

ANAESTHESIA PLAN AND RECORDS – DECAPOD CRUSTACEAN

Animal information

Animal name/ID:______________________Responsible/Tutor:____________________________________
Species:_______________________________Sex:______Weight(g):_______________Age:______________
Health condition:____
Premedication:_____________________________Dose:____________Rote:________Lenght:____________
Particularity:____

Anaesthesia Plan

Procedure:____Expected duration:__________________
Anaesthesia depth:________________Risk: _______see note_______Drug:________________________
Dose:____________________Volume:_____________________Route:___________________________________
Environment: water /room T:_________ ARH:_________water hardness:_________water pH:________
Time of Remove from water:______________________Return to water:____________________________

Anaesthesia Monitoring

Time of Procedure start: ______________ Induction:___________________Sedation: ______________
Anaesthesia: _________________ Procedure end:______________ Initial recovery:_________
Full recovery:________________ Non-responsive: ______________________________________
Quality of Induction:_______________________________ Recovery:_____________________________
Complications____

Time	Equilibrium	Response to stimuli	Antennae withdraw	Limb withdraw	Limp body	Mobility	Ventilatory function	Heart rate
Hour	Present Absent	Present Absent	Present Absent	Present Absent	Present Absent	Present Absent	Preserved Arrest	BPM Acardia

Post Anaesthesia Care

Clinical assessment:____
Recommended care:____
Additional comment:____

ARH: air relative humidity
BPM: beats per minute
ID: identification
T: temperature

Risk: 0 = low
1 = low-medium
2 = medium
3 = medium – high
4 = high

Practitioner signature
____
Practitioner name
Anaesthetist
