# Supplementary material for: Anaesthesia of decapod crustaceans
Source: Vet Anim Sci. 2022 May 14;16:100252. doi: 10.1016/j.vas.2022.100252 (PMC9127210; doi:10.1016/j.vas.2022.100252)
Supplement: Supplementary file 2 [file mmc2.pdf]

Record № _______________

ANAESTHETIC ACCOUNTABILITY RECORD

Commercial name:____ Active principle:____

Form:____ Strength:____ Expire date:______/______/______

Date	Purpose of use	Species	Initial amount	Amount used	Final amount	Amount wasted	Name	Signature	Comments
